# Sub-Nanometer Accuracy Combination Processing Technology for Nickel–Phosphorus Modified Surfaces Based on Aluminum Reflector Mirror

**DOI:** 10.3390/mi13040560

**Published:** 2022-03-31

**Authors:** Hao Hu, Chao Xu, Tao Lai, Qilin Yang, Xiaoqiang Peng, Junfeng Liu, Yupeng Xiong, Jia Qiu

**Affiliations:** 1 Laboratory of Science and Technology on Integrated Logistics Support, College of Intelligence Science and Technology, National University of Defense Technology, Changsha 410073, China; tiny_hh@139.com (H.H.); wjsxcr@126.com (C.X.); qilinbit@163.com (Q.Y.); ljf20090702122@163.com (J.L.); bear_xiong@163.com (Y.X.); 2 Hunan Key Laboratory of Ultra-Precision Machining Technology, Changsha 410073, China; 3 College of Intelligence Science and Technology, National University of Defense Technology, Changsha 410022, China; qiujia@163.com

**Keywords:** Ni–P modified layer, processing technology, MRF, metal reflector mirror, sub-nanometer accuracy

## Abstract

The surface of metal mirrors is often polished by electroless coating with a Ni–P modified layer after single-point diamond turning. In practice, however, improvements in mirror quality are closely related to the polishing environment, polishing medium, and polishing force. If not adequately controlled, processing defects such as visible scratches can lead to the deterioration of surface roughness. Based on the Ni–P modified surface of a metal reflector mirror, this study optimizes the configuration of magnetorheological figuring (MRF) fluid and polishing process parameters so that MRF high-efficiency surface modification can be realized and the scratch problem can be resolved. The processing method of a high-performance metal mirror is developed by studying the high-efficiency and high-precision processing technology based on small head smoothing. The surface roughness achieved by the proposed method was better than Ra = 0.39 nm. The ultrasonic cleaning process effectively improved the surface roughness after processing. According to the combined processing technology developed in this study, the modified layer of the parabolic mirror with a diameter of 370 mm was processed, and the surface quality was increased from RMS = 338.684 nm to RMS = 21.267 nm.

## 1. Introduction

Aluminum alloy materials are inexpensive, have low density and high strength, and are easy to process and manufacture. Metal mirrors are rarely used in visible or ultraviolet systems due to ultraprecision fabrication difficulties. The surface after single-point diamond turning (SPDT) can be directly used in the imaging applications of infrared optical systems [1,2]. However, the soft texture of the aluminum alloy surface and its susceptibility to scratching are also apparent, thus limiting its application in the visible light field. Modification on the substrate surface is a common method to improve the optical performance of the mirror surface [3,4]. Ni–P alloys are an excellent modified layer material that is widely used in the aviation and defense industries [5,6].

Chemical coatings on Ni–P alloys can improve the surface quality of mirrored substrates via surface modification and processing [7,8]. Ni–P layers enable several processing techniques (e.g., SPDT, chemical mechanical polishing (CMP), magnetorheological finishing (MRF), and ion beam figuring) for reducing the roughness of the optical surface).

After SPDT [9], the surface of the Ni–P alloy modified layer can be optically polished to improve the surface quality and remove residual tool marks. The Ni-P layer is processed using SPDT with an accuracy of 60 nm in root mean square (RMS) analysis, and the surface roughness reaches Ra = 4.157 nm [10]. Kinast et al. polished Ni–P alloy using aluminum 6061 as the experimental material matrix. After ultraprecision turning, a layer of electroless nickel coating was modified on the mirror surface, and the processing accuracy of optical elements was then improved by processing the nickel coating layer [11]. The results showed that the electroless Ni–P modified layer was more uniform and had fewer surface defects compared with the electroplated nickel layer. A surface roughness of less than RMS = 1 nm was obtained [12].

Namba et al. studied the processing of Ni–P alloy using CMP. Under controlled variables, a surface roughness of less than Ra = 0.3 nm was obtained by processing the material with nanosized silica polishing abrasive particles and flannel polishing disks [13]. Lei Hong et al. processed Al-based Ni–P alloy by chemical mechanical polishing. The experimental workpiece was polished with silica polishing abrasive particles and ferric oxide ions as the oxidant [14,15,16]. A surface quality of Ra = 3.73 nm was obtained by processing SiO_2_/Fe_2_O_3_ composite abrasive particles on Ni–P alloy materials, and a surface quality of Ra = 0.48 nm was obtained by using SiO_2_/CeO_2_ composite abrasive particles with a core-shell structure. Salleh Sideq compared the polishing performance of spherical and nonspherical colloidal silica abrasive particles in the Ni–P alloy polishing experiment [17]. The results showed that the nonspherical colloidal silica abrasive particles did not produce defects on the surface of the workpiece and were superior to the spherical silica abrasive particles, leading to a surface quality of Ra = 0.756 nm. Zhang et al. developed a novel environment-friendly CMP slurry, consisting of silica, hydrogen peroxide (H_2_O_2_), malic acid, and deionized water [18]. The surface roughness Ra and peak-to-valley (PV) values were 0.44 and 4.49 nm, respectively, with an area of 71 μm × 53 μm.

An MRF process for the post-treatment of diamond turning is presented to remove the periodic microstructures and subsurface damages with improvement of the original figure and surface roughness by Jeon [19]. Aibo of Changchun University used MRF technology to polish aspheric mirrors with an aspheric aperture of 255 × 152 mm; the RMS value of the initial surface shape was 124.01 nm, and the RMS value of the finished surface shape was 15.19 nm. The residual lines after turning were removed, and the surface roughness was Ra 2.62 nm [20]. The experimental results indicated that MRF is suitable for removing periodic micropatterns caused during the SPDT process with the progress of the original figure and surface roughness. A variety of materials, such as Zerodur, plastics, and hard polycrystals, were examined before high-quality figuring and finishing very well with MRF [21,22]. However, investigations about the effect of MRF polishing fluids’ composition on the Ni-P layer polishing performance, such as the material removal rate, surface roughness, and cleanliness, were rarely reported.

The properties of the modified layer and the matrix are quite different due to the different material compositions. Furthermore, different proportions of nickel and phosphorus in the modified layer will also significantly change the properties of the modified layer, thus affecting physical properties, such as hardness, elastic modulus, and reflectivity, and in turn subsequent processing [12]. Therefore, it is critical to fully understand the surface characteristics of the modified layer. A suitable processing route should be studied according to surface characteristics. Otherwise, surface defects such as scratches will appear during processing of the modified layer, which reduce the strength and service life of the modified layer.

Much research has been conducted on the polishing of the modified layer, but further research is needed on how to stably control scratches during processing, how to achieve high-precision and efficient processing on the modified surface of the metal mirror with a complex curved surface, and how to improve the surface roughness of the modified layer.

## 2. Materials and Methods

### 2.1. Materials

The following materials were applied in this study: aluminum alloy (Microhension No. 6061/A96061) and nickel–phosphorus alloy (Ni–P) (the quality fraction of Ni was 87.5 wt%; the quality fraction of P was 12.5 wt%, manufactured by Guang Zhou Yi Shun Chemical Co., Ltd., Guang Zhou, China). The aluminum alloy was used as the base material of the workpiece, and the alloy Ni–P was used as the surface coating layer. There were four kinds of abrasive particles: cerium oxide (CeO_2_), silicon dioxide (SiO_2_), alumina (Al_2_O_3_), and diamond (C).

The nickel crystal has a face-centered cubic structure, 12 nickel atoms around each nickel atom. The existence of phosphorus in the modified layer will affect the grain size and thus the structure of the modified layer. In addition, different heat treatment processes will also affect the structure of the modified layer. Aluminum alloy is used as the base material of electroless nickel coating, and the zinc-dipping premodified layer method is a mature and reliable modification process [23,24,25,26,27]. Operational procedures include the formation of base parts, degreasing treatment, etching, zinc dipping, nitric acid stripping, secondary zinc-dipping treatment, alkaline condition chemical precoating, acidic condition chemical coating, baking, and analysis of finished products.

In the subsequent modification process, the Ni–P modified layer with excellent properties can be obtained by reasonably regulating the reaction conditions and rate according to the reaction mechanism. It should be noted that aluminum is an excellent material for nickel coating, and when the substrate is changed, the actual process will also have significant problems. Therefore, it is necessary to improve that plate process to obtain an excellent modified layer. Furthermore, we should pay attention to the thickness of the modified layer. When the modified layer is too thin, the modified layer will be damaged in the turning process and even seriously damage the diamond tool, which will bring significant problems to the subsequent processing.

### 2.2. SPDT Turning Process of the Modified Layer

**Figure 1:**
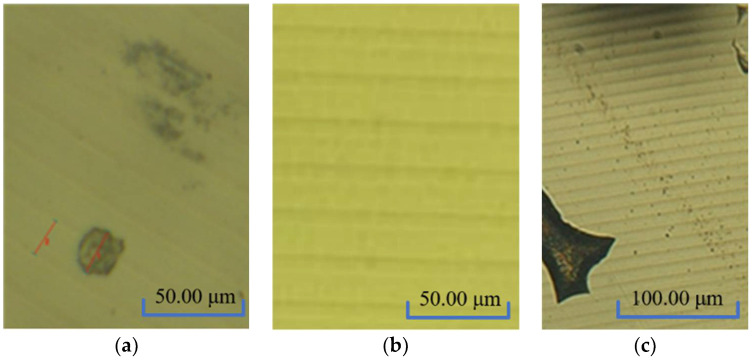
Surface morphology after turning. (**a**) Substrate-turning lines. (**b**) Modified-layer-turning lines. (**c**) Modified-layer-turning damage.

The unmodified surface is an aluminum alloy matrix with a soft texture. After processing, there are many impurities in the material due to the matrix itself, so random defects will appear after turning, as shown in Figure 1a. In contrast, the surface of the modified layer is better than that of the unmodified metal mirror because of uniform coating. Therefore, there are no random defects on the processed surface (Figure 1b). The total thickness of the modified layer is about 80–100 μm, and the modified layer is damaged by the large removal amount, as shown in Figure 1c.

Both the modified layer and the unmodified substrate have periodic lines after turning, and the reflection grating structure, such as periodic turning lines, will bring dispersion and ghosting problems to the mirror. Dispersion will cause the metal mirror surface to generate the “rainbow” phenomenon after SPDT, which will affect the subsequent processing and the usage of a mirror. Problems such as dispersion and ghosting observed by naked eyes are caused by the optical characteristics of plane reflection grating. The plane reflection grating [28,29] is calculated by Equation (1):

(1) $$d(\sin\theta_{i}+\sin\theta_{m})=\lambda ,\;m=[\pm (0,1,\;2,3\ldots )]$$

where *d* is the grating constant, *θ_m_* is the diffraction angle, *θ_i_* is the incident angle, and m is the diffraction order.

Different ultraprecision turning parameters will result in different surface shape errors of optical parts after processing. For the surface shape error, the traditional evaluation indexes mainly include PV and RMS values, but the error information contained in these indexes is quite limited. Power spectral density (PSD) can be used to evaluate the surface error and analyze the wavefront error information contained in the profile, especially the midhigh frequency error information. The one-dimensional PSD function is expressed by Equation (2) [30]:

(2) $$PSD(f)=\int_{0}^{2\pi }PSD(f_{x},f_{y})d\sqrt{{\left(\cos\theta /L_{x}\right)}^{2}+{\left(\sin\theta /L_{y}\right)}^{2}}$$

where *L_x_* represents horizontal data sampling, *L_y_* represents the data sampling length in the vertical direction, and the unit of a one-dimensional wavefront PSD function is nm^3^.

### 2.3. MRF Process of the Modified Layer

Cerium oxide polishing powder is widely used in polishing, and the MRF fluid prepared by cerium oxide polishing powder has good properties and has a good effect on processing materials, such as fused silica and microcrystals. However, visible scratches will occur when processing the Ni–P modified layer when cerium oxide polishing powder is used as abrasive particles to prepare MRF fluid.

**Figure 2:**
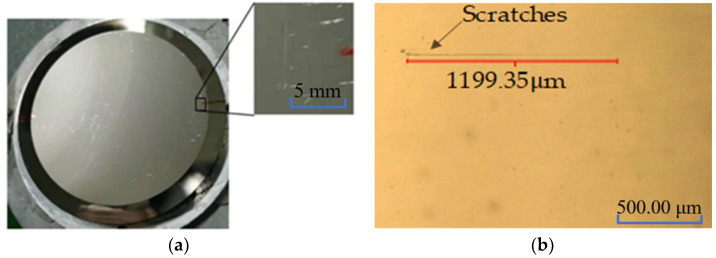
Scratch phenomenon. (**a**) Scratch phenomenon. (**b**) Scratches measured by a microscope.

Cerium oxide is a rare-earth metal oxide and belongs to the cubic system with a polygonal structure. Cerium oxide is an excellent abrasive particle for its face-centered cubic structure, moderate crystal grain size, and three levels of edges and corners; the chemical and mechanical actions coincide in MRF. The surface quality is also good with the abrasive particle, but visible scratches are produced on the surface when processing the Ni–P modified layer of the mirror, as shown in Figure 2 (VHX-600E Super Depth of Field Microscope manufactured by Keyence Corporation of Japan).

The process of MRF is influenced by many aspects, such as material characteristics, MRF fluid properties, and polishing process parameters. By studying the influence of a removal function on a processing effect, the material removal mechanism in the process of MRF is analyzed. The hydrodynamic pressure of polishing abrasive particles is mainly analyzed, but the influence of gravity and magnetic field on the polishing abrasive particles is ignored.

(3) $$F_{r,n}=\frac{1}{2}H\pi x_{2}\delta$$

(4) $$F_{r,t}=\gamma HA_{p}$$

(5) $$A_{p}(\delta )=\frac{x_{1}^{2}}{4}\sin^{-1}\frac{2\sqrt{\delta (x_{1}-\delta )}}{x_{1}}-\sqrt{\delta (x_{1}-\delta )}(\frac{x_{1}}{2}-\delta )$$

**Figure 3:**
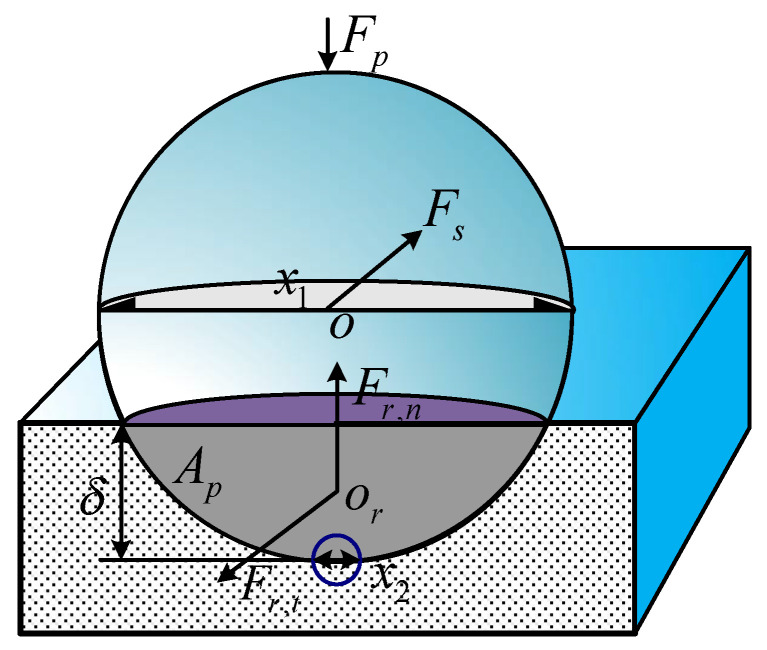
Theoretical calculation model of effective indentation depth of abrasive particles.

The “double-edged circle” model shows that the polishing process of the processed material by abrasive particles can be regarded as the sliding stamping process of a half-space by a rigid indenter, as shown in Figure 3 [31]. Here, *x*_1_ is defined as the equivalent diameter of the polishing abrasive particles, *x*_2_ is the diameter of the edge circle where the polishing abrasive particles contact the plane of the processed material, and H represents the Vickers hardness of the surface of the processed material. Δ represents the depth of polishing abrasive particles pressed into the plane of the processed material, and *γ* is the resistance coefficient of the material. The tangential projection area *A_p_*, tangential resistance *F_r,t_,* and normal resistance *F_r,n_* of abrasive particles pressed into the workpiece can also be obtained.

The contact state between abrasive particles and the workpiece surface is also analyzed. During the process of MRF, a single abrasive particle is mainly subjected to hydrodynamic pressure *F_p_*, shear force *p*, and resistance *F_r_* in which *F_p_* is the normal resistance *F_r,n_* of a single abrasive particle. The resistance mainly comes from the contact and friction between abrasive particles and the workpiece surface. According to the theory above, the load and shear force on a single abrasive particle are shown in Equations (6) and (7) [31]:

(6) $$F_{p}=\frac{1}{4}p\pi x_{1}^{2}$$

(7) $$F_{s}=\frac{1}{4}\tau \pi x_{1}^{2}$$

By transforming the above equations, the depth of abrasive particles pressed into the workpiece can be obtained, as shown in Equation (8):

(8) $$\delta =\frac{2F_{p}}{H\pi x_{2}}$$

The hardness of cerium oxide is equivalent to that of the Ni–P modified layer. When *F_s_* ≤ *F_r,tmin_*, the shearing force generated by processing is insufficient to overcome the minimum tangential resistance of abrasive particles, and the abrasive particles will roll continuously, thus resulting in visible scratches. In addition, the grating track is applied in MRF processing, and there is greater pressure on the processing track; thus, the scratches often appear on the grating track.

### 2.4. CCOS Smoothing Process of the Modified Layer

**Figure 4:**
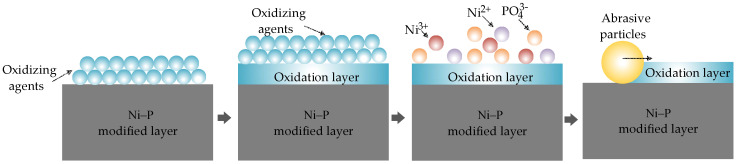
Principle of chemical reaction.

The removal mechanism of nickel-plated aluminum mirror material was analyzed at the interface chemistry level from the perspective of balance between chemical action and mechanical action in the smoothing process. The critical role of chemical reactions, such as redox reactions in the smoothing process was considered, and the corresponding smoothing removal model was established. A thin oxide film is formed on the surface of the Ni–P alloy workpiece under the action of a smoothing fluid when an oxidizing agent, such as hydrogen peroxide, is used in the smoothing process. The removal process is shown in Figure 4.

A thin oxide layer is formed on the surface of the Ni–P alloy workpiece when an oxidant is used in the smoothing process. This consists of NiO, Ni_2_O_3_, and P_2_O_3_. The oxide layer is loose and can be easily removed under the mechanical action of smoothing abrasive particles, and a small surface roughness is obtained [23,24,25,26,27].

(9) $$P_{2}O_{3}+H_{2}O\to H_{3}PO_{4}$$

(10) $$NiO+H_{2}O⇄Ni{\left(OH\right)}_{2}⇄Ni^{2+}+OH^{-}$$

(11) $$Ni_{2}O_{3}+H_{2}O⇄Ni{\left(OH\right)}_{3}⇄Ni^{3+}+OH^{-}$$

These substances are hydrolyzed during processing, as shown in Equations (9)–(11). Under hydrolysis, nickel and phosphorus elements change from a solid state to a liquid state; nickel oxides react with water to generate nickel hydroxide. In Equations (9) and (10), we see that Ni(OH)_2_ and Ni(OH)_3_ are consumed in the reversible reaction. The reversible reaction continues to go to the right of the equation, and nickel is continuously converted into Ni^2+^ and Ni^3+^. This step promotes the dissolution of nickel oxide and ensures that new surfaces are constantly exposed during the reaction.

### 2.5. Cleaning Process of the Modified Layer

**Figure 5:**
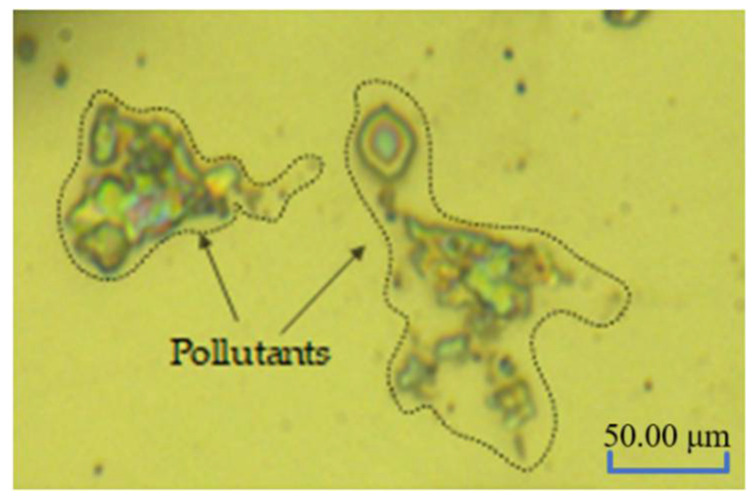
Surface contamination after processing.

After processing, the mirror modified layer is affected by processing media, such as particles in polishing fluid. Many pollutants remain on the surface of the processed and modified layer, which will affect its service performance (Figure 5).

The surface of the processed metal mirror is cleaned to maintain a modified layer with good smoothness. Physical, chemical, or mechanical methods to remove the pollutants adsorbed on the surface can achieve good results.

Alcohol scrubbing is a cheap and efficient cleaning method to clean the reflector mirror surface. Scrubbing is a type of contact cleaning that uses a clean cloth to contact the surface of the aluminum mirror dripping with alcohol and uses the drag force of liquid to take pollutants away from the surface of the aluminum mirror. Zhang et al. showed this phenomenon during the scrubbing process [32,33]. The Stokes drag force on cleaning fluid on the surface of the aluminum mirror is expressed by Equation (12):

(12) $$F_{D}=5.1\pi \eta d_{p}v_{x}$$

Here, *η* is the viscosity of the liquid, *d_p_* is the particle diameter of the pollutant, and *v_x_* is the fluid velocity between the center of the pollutant and the surface of the aluminum mirror.

Therefore, larger pollutant particle sizes lead to a greater liquid drag force. This makes it easier to remove the pollutants on the surface of the aluminum mirror. The polished aluminum mirror is rinsed with deionized water and gently wiped with alcohol. The pollution components on the surface of the aluminum mirror can be largely removed.

Noncontact cleaning methods, such as ultrasonic cleaning, are used in addition to the contact cleaning represented by deionized water and alcohol in daily use. The principle of ultrasonic cleaning is that ultrasonic waves vibrate the medium liquid, and energy is applied to the pollutant particles, as shown in the equation below; this energy plays a role in cleaning the workpiece [33].

(13) $$W\propto Af^{2}d^{2}$$

Here, *f* is the cleaning frequency, and *d* is the diameter of pollutant particles. The energy obtained by the pollutants can vibrate away from the surface of the aluminum mirror; larger particles lead to larger energy. This makes it easier to remove the pollutants on the surface of the aluminum mirror.

## 3. Experiment

### 3.1. SPDT

Reflectivity is an important optical characteristic of optical elements and an evaluation index of its light transmission ability; the size is less than 1. The reflectivity changes with a different process. Besides the influence of the material itself, the surface microstructure will also lead to a loss of light energy and information. Generally, the deterioration of surface roughness is the leading cause of scattering loss. The sample turning parameters are shown in Table 1.

**Figure 6:**
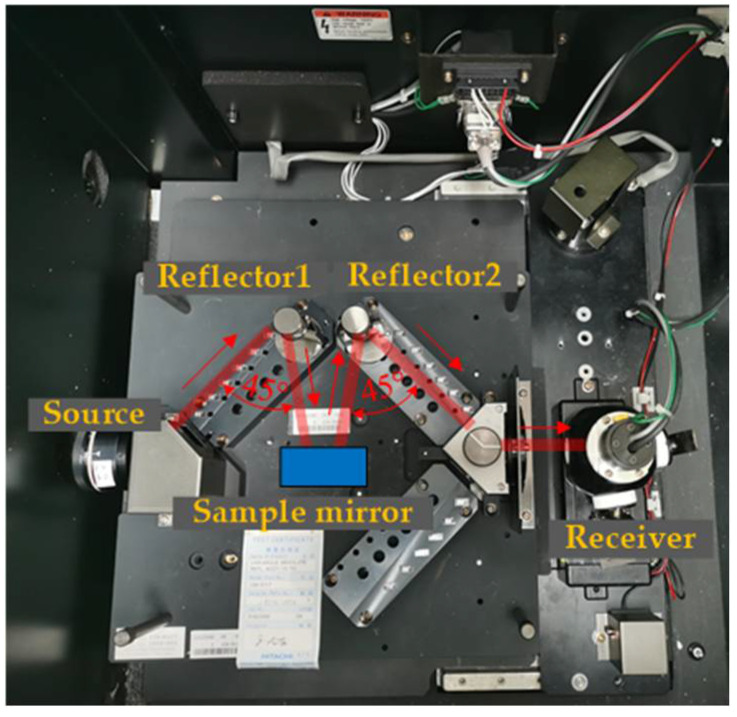
Experimental test of modified layer spectrophotometer.

The instrument used here is a Hitachi U-4100 spectrophotometer (Tokyo, Japan) and is used to measure reflectivity. Figure 6 shows that its measurement range is 240–2600 nm. The sample mirror to be tested is placed on the stage in the sample bin with a reflection angle of *θ_f_*. The stage and receiver are rotated with a reflection angle *θ_f_*, and the energy signal of the sample is thus obtained. The metric monitored is the reflectivity of the sample at the reflection angle *θ_f_*.

### 3.2. MRF

Analysis of the abovementioned double-edged circle model shows that the indentation depth δ will be in a smaller range when abrasive particles with higher hardness are used. Cerium oxide polishing powder has low hardness. To avoid scratches, it is necessary to reduce the rotating speed of the polishing wheel, which significantly influences MRF processing. Therefore, different polishing abrasive particles, such as silica, alumina, and diamond, were selected. The different abrasive particles are configured into MRF fluid to explore the possibility of MRF processing of the modified layer.

**Figure 7:**
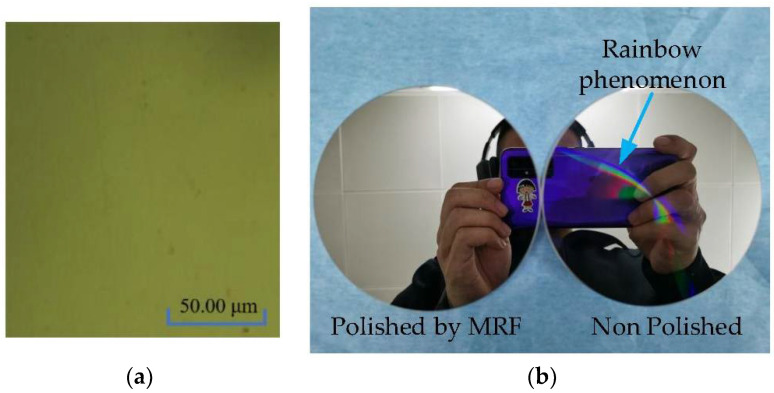
Polishing of modified layer to eliminate dispersion phenomenon. (**a**) Scratch phenomenon is suppressed; (**b**) the dispersion phenomenon is eliminated after polishing.

MRF fluid plays a vital role in MRF, and its performance directly affects the success of the MRF process. Common polishing abrasive particles are cerium oxide, silicon dioxide, aluminum oxide, and diamond in the order of increasing hardness. Polishing abrasive particles with hardness higher than cerium oxide are used to configure the corresponding MRF fluid, and the modified layer is processed by MRF. When the hardness of polishing abrasive particles is increased, the pressing depth of abrasive particles on the machined surface can be relatively reduced under the condition of achieving the same shearing force. The result shows that abrasive particles with higher hardness will significantly suppress the scratch phenomenon that occurred before. The processed surface is shown in Figure 7.

### 3.3. CCOS

The CCOS smoothing fluid can transport the smoothing abrasives during processing and take the products removed by smoothing away from the processing surface; thus, the fluid plays a specific cleaning role. In the configuration of smoothing fluid for the small head of a Ni–P modified layer, the abrasive silica particles are usually spherical with a large specific area, low viscosity, good dispersibility, and easy cleaning. Moreover, the particle size can be controlled to the nanometer level, and it has better particle size dispersion than cerium oxide and aluminum oxide; thus, nanosilica is selected as the CCOS-smoothing abrasive.

The use of an oxidant can promote the chemical reaction during processing, and its selection standard is to make the removal process balance chemical and mechanical action. In the smoothing process, when the material removal amount is small, the surface quality after processing will be affected if the oxidant is too strong. Hydrogen peroxide is a suitable option, and it offers easy cleaning and is suitable for chemical mechanical smoothing. The generation of an oxidant is necessary. When no oxidant is used during processing, the smoothing abrasive particles are in direct contact with the surface of the modified layer, thus resulting in low smoothing removal efficiency and difficulty in improving the surface roughness.

The pH value of the smoothing fluid has a crucial influence on the quality after smoothing. Using sodium hydroxide fluid and organic acid to adjust the pH value of the fluid can inhibit the crystallization of abrasive silica particles in the smoothing fluid. However, complexation of metal ions and organic acid can improve the mechanical removal of materials. Component parameters of the smoothing fluid are shown in Table 2.

An asphalt-smoothing disk and configured smoothing fluid can smooth the modified layer with a small head. The smoothing pressure is set to 0.03 MPa, and the smoothing fluid with the disk is in contact with the processed surface under the air pressure. The surface smoothing experiment of the modified layer is performed via the grating trajectory. 

## 4. Results

### 4.1. Surface Quality after SPDT

**Figure 8:**
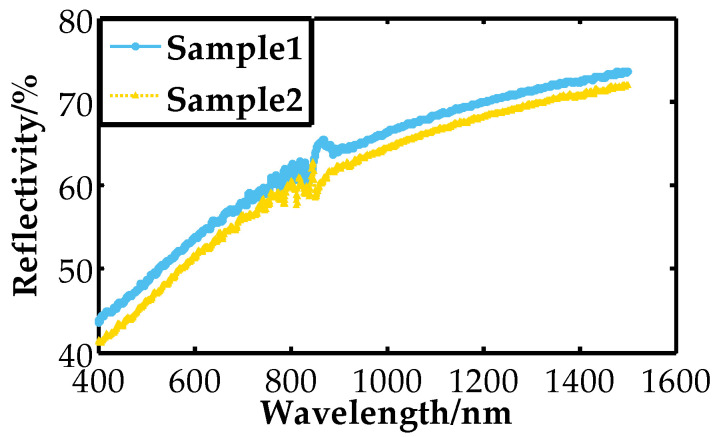
Reflectivity of Ni–P modified layer after different turning processes.

The total reflectivity of the sample mirror at a reflection angle *θ_f_* in band B (b*_i_*~b*_j_*) is half of the total reflectivity of light S and light P as shown in Equation (14). The reflectivity of sample 1 is obtained with SPDT (the parameters shown in Table 1). The cutting depth becomes larger with increasing spindle speed, and the reflectivity of sample 2 is obtained. The reflectivity of two nickel–phosphorus-modified plane mirrors with different turning processes was also measured (Figure 8).

(14) $$F^{Z}(f_{i}^{Z}\sim f_{j}^{Z})=\frac{1}{2}[F^{S}(f_{i}^{Z}\sim f_{j}^{Z})+F^{P}(f_{i}^{P}\sim f_{j}^{P})]$$

The reflectivity ratio of surface diffuse reflection to total reflection is total integrated scattering (TIS), which can be approximately defined as [34]:

(15) $$TIS\approx {\left(\frac{4\pi \sigma }{\lambda }\right)}^{2}$$

**Figure 9:**
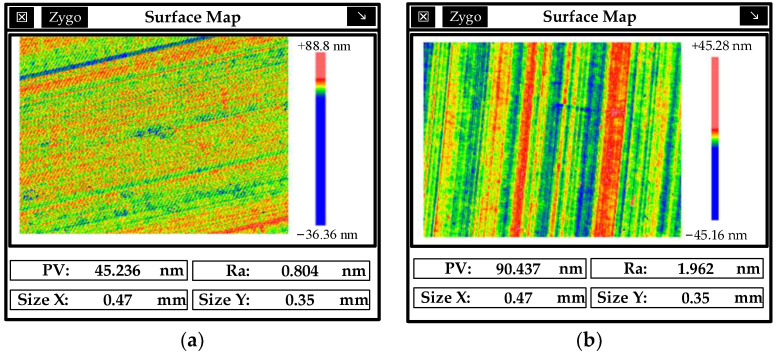
Surface roughness of the modified layer after turning. (**a**) Surface roughness of modified layer sample 1. (**b**) Surface roughness of modified layer sample 2.

The term *σ* is root mean square surface roughness, and *TIS* is directly determined by the ratio of root mean square surface roughness to light wavelength. A larger *TIS* value implies a larger random roughness of the surface. In some cases, *TIS* is used to calculate the surface roughness back, which shows that random surface roughness is critical in specular reflectivity. Therefore, the reflectivity of the two plane mirrors was shown in Figure 8, and the surface roughness values are shown in Figure 9 below.

Figure 9 shows that the surface roughness of the modified layers is better than Ra = 2 nm after different ultraprecision turning processes. As a result, the improvement of surface roughness can improve the reflectivity. By analyzing the reflectivity curve, we see that the reflectivity of the modified layer in the infrared band exceeds 70% without coating treatment. The reflectivity keeps dropping with shorter wavelengths.

### 4.2. Surface Quality after MRF

**Figure 10:**
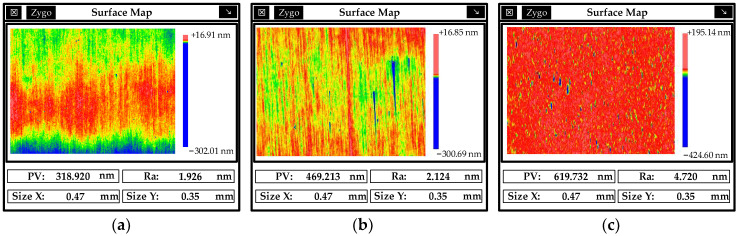
Surface roughness of modified layer after MRF processing with different abrasive particles. (**a**) Polished roughness with silica, (**b**) polished roughness with alumina, (**c**) polished roughness with diamond.

Both “dispersions” and “ghosts” can affect mirror image quality. The subsequent polishing process can effectively remove turning lines, plane reflection grating, dispersion, and ghosting. Figure 9b shows that the polished mirror (left) contrasts with the unpolished mirror (right), and the “rainbow phenomenon” is effectively removed. Silica, alumina, and diamond polishing abrasive particles can configure the corresponding MRF fluid. The surface of the modified layer is polished by MRF, and the polished surface roughness is shown in Figure 10.

Ra increases with increasing hardness of polishing abrasive particles. Noticeable deep pits appear when alumina is used as polishing abrasive particles, and PV = 469.213 nm. The abrasive particles will be embedded in the surface of the workpiece and dragged along with the workpiece when the hardness of polishing abrasive particles is too high and the diameter x*_2_* of the edge circle is too large. At the same time, the surface energy of nano-alumina is relatively high, and easy agglomeration of particles will also increase the surface roughness after processing. The mirror surface has a tail–tail phenomenon when diamond is used as a polishing abrasive particle. This significantly worsens the surface roughness. From a surface roughness point of view, silica was selected as polishing abrasive particles to configure the MRF fluid; it was then optimized and improved.

**Figure 11:**
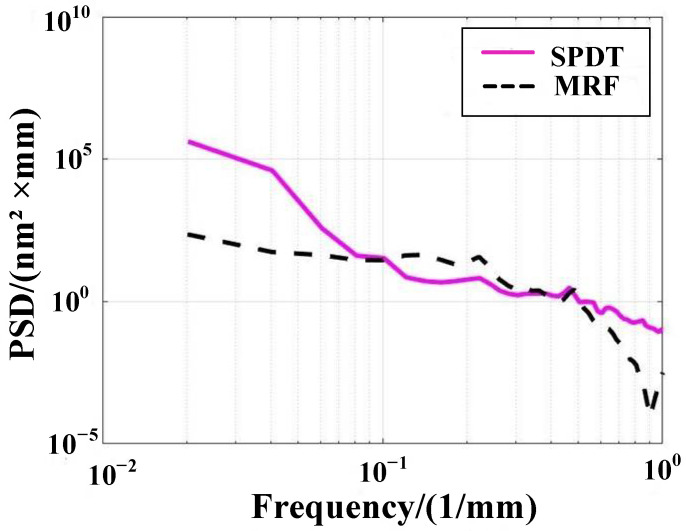
PSD Analysis of SPDT and MRF machining.

Although MRF can remove the periodic knife lines generated in SPDT processing, MRF will deteriorate the quality of the mirror surface and cannot effectively remove the intermediate frequency error, as shown in Figure 11. Therefore, follow-up processing is required to improve the surface quality. 

### 4.3. Surface Quality after CCOS

**Figure 12:**
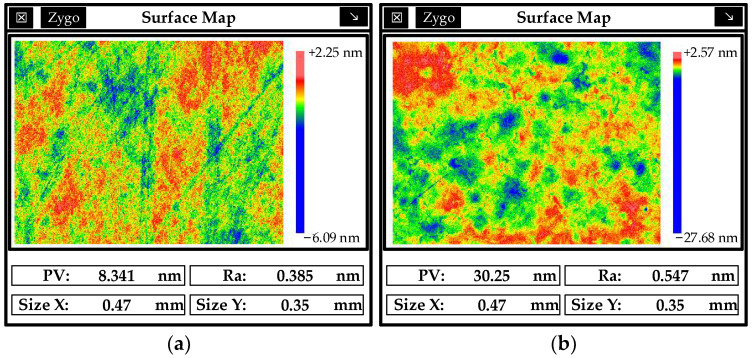
Influence of smoothing process optimization on surface roughness. (**a**) Surface roughness optimized by smoothing process; (**b**) surface roughness with too strong chemical action of smoothing fluid.

In the CCOS smoothing process, the type, particle size, and pH value of the fluid will directly affect the surface quality. The Ra is 0.55 nm. The balance between mechanical and chemical action is important in the reaction. When the smoothing fluid is excessively oxidizing, pits will appear on the processed surface that worsen the surface roughness (Figure 12b).

**Figure 13:**
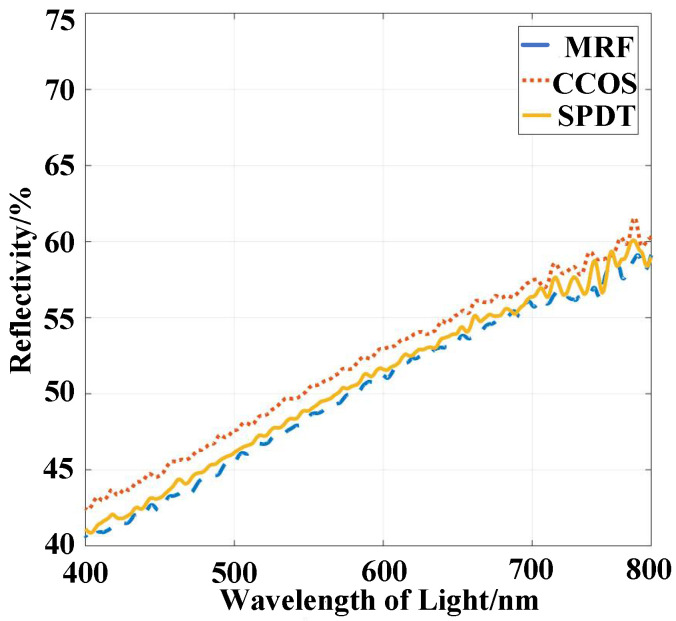
Visible light band reflectance comparison of sample 1.

The reflectivity of the modified layer in each processing process was measured. The experimental results are shown in Figure 13. The roughness will deteriorate after the MRF and improve after the CCOS. Therefore, the reflectance decreases after MRF and returns to the previous level after CCOS.

### 4.4. Surface Quality after Ultrasonic Cleaning

**Figure 14:**
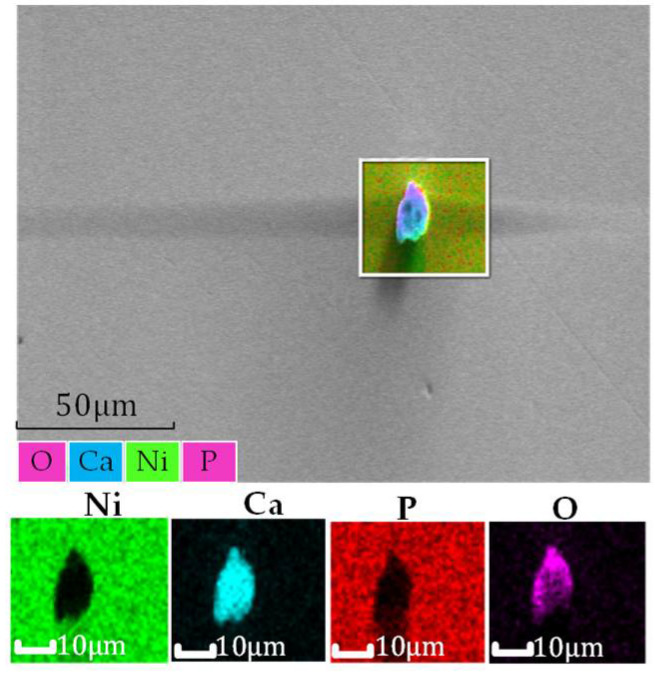
Measurement of impurities on the surface of a modified layer.

Many impurity particles will be adsorbed on the surface of the modified layer after smoothing. Therefore, the elemental on the surface of the uncleaned modified layer is analyzed after processing, as shown in Figure 14 (EVO MA 10/LS 10 Scanning Electron Microscope from Carl Zeiss, Germany).

The results show that the uncleaned surface after processing contains impurity elements except for nickel and phosphorus.

**Figure 15:**
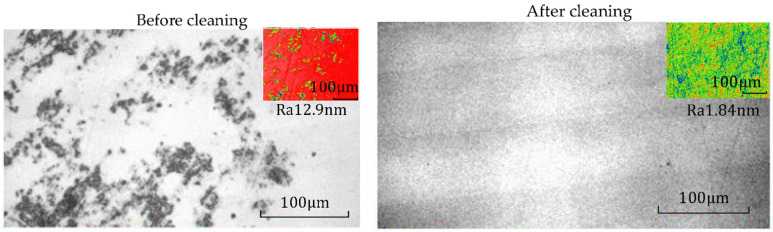
Surface roughness of a modified layer before and after ultrasonic cleaning.

The roughness of the modified layer before and after cleaning is shown in Figure 15, and good surface roughness was obtained after cleaning. Therefore, the cleaning process is necessary to improve the surface roughness in the combined processing technology.

## 5. Discussion

**Figure 16:**
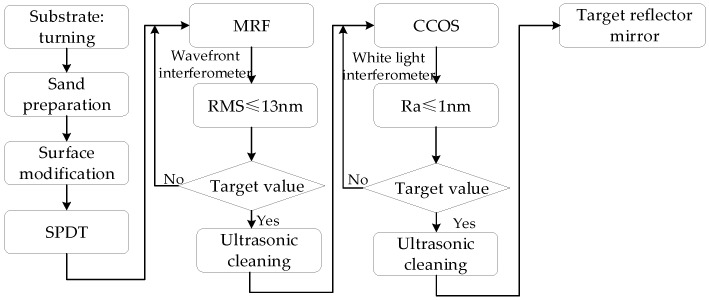
Road map of combined processing technology for Ni–P modified layer.

The manufacturing process of metal mirrors mainly includes turning, polishing, and post-treatment. After the coating of the modified layer is finished, ultraprecision turning is first used to obtain a better surface quality to enter the polishing process. Then, the surface roughness is improved to meet the final processing requirements after MRF and CCOS, as shown in Figure 16.

(1) NC rough processing: According to the technical index requirements of the target mirror, the mirror substrate is turned by the NC with 6061 aluminum alloy. When the surface roughness Ra value is 2 μm, an electroless nickel coating is performed to obtain the blank of the metal mirror-modified layer.
(2) Ultraprecision turning: To improve the surface quality, the metal coating mirror blank is turned by the SPDT with the appropriate parameters to then clean the surface. The wavefront interferometer and white light interferometer are used to measure the processed surface to prepare for the following process.
(3) MRF: The coating mirror is polished by the MRF based on the MRF removal function of the modified layer and the measured initial surface figure. After processing, the surface is cleaned, and the wavefront interferometer is used to measure the surface. If the surface accuracy requirements are not met, then the coating mirror is modified by MRF iteration until the surface accuracy requirements are met.
(4) Surface quality improvement: First, an appropriate CCOS polishing disk and polishing fluid need to be ensured. The MRF-polished mirror is then smoothed by the CCOS with a small head. After cleaning the surface, the white light interferometer is used to measure the surface quality. If the surface quality requirements are not met, then mirrors are iteratively smoothed until the high surface quality requirements are achieved. During the smoothing process, the amount of processing removal should be focused to prevent the surface from being significantly degraded.

The above-mentioned combined processing technology can eliminate turning lines and obtain high-quality surface quality while performing high-precision deterministic modifications. If the turning surface accuracy does not meet the requirements, then repeated iterations of MRF-polishing can be performed. Similarly, if the CCOS process improves the surface roughness but does not meet the requirements, then an MRF iteration can be performed. Finally, the surface accuracy and surface quality can meet the requirements.

**Figure 17:**
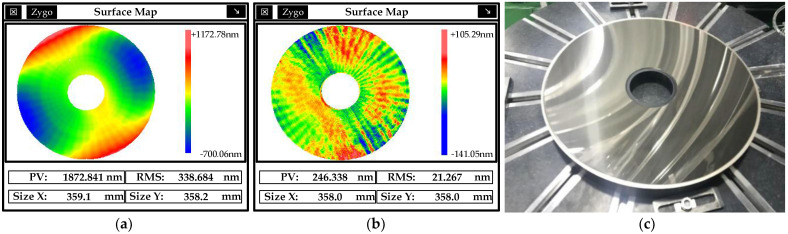
Results of paraboloid mirror before and after processing. (**a**) Before processing, (**b**) after processing, (**c**) physical processed mirror.

The modified layer of the parabolic mirror with an aperture of 370 mm and a vertex curvature radius of R_0_ = 1/*c* = −196.7 mm was processed. After coating and ultraprecision turning, the RMS of mirror surface reached 338.684 nm, and the parabolic reflector measurement result is shown in Figure 17a. After MRF polishing and processing, the surface accuracy of the parabolic mirror was increased from 1872.841 nm PV/338.684 nm RMS to 246.338 nm PV/21.267 nm RMS; the surface roughness was 0.61 nm Ra. The physical processed parabolic mirror is shown in Figure 17c.

## 6. Conclusions

This study examined a combined processing technology for ultraprecision machining of modified layers of metal mirror modified layers. The SPDT turning, MRF polishing, and CCOS smoothing techniques were used to improve the surface quality of ultraprecision machining of the modified layer of metal mirror. The initial surface shape and quality obtained by SPDT turning achieved a mirror effect, but after turning, the mirror surface formed periodic cutting lines, which were modified by MRF. Finally, a high surface quality was obtained by the CCOS smoothing process.

With the proposed strategies, machining precision of metal mirror optics can be further improved, which will promote the application of metal mirror optics in visible light band as well as the application of SPDT turning, MRF polishing, and CCOS smoothing in the field of metal mirror optic fabrication.

## Figures and Tables

**Table 1:** Turning parameters of SPDT.

Turning Parameters	Spindle Speed (rpm)	Feed Rate (mm/min)	Depth of Cut (μm)
Sample 1	1200	5	1.4
Sample 2	1500	10	2.0

**Table 2:** Composition parameters of the smoothing fluid.

Smoothing Fluid Parameter	Abrasive Particle Size	Abrasive Particle Concentration	Oxidant Composition	Oxidant Concentration	pH Value
Parameter value	50 nm	5%	H_2_O_2_	1%	4

## Data Availability

Not applicable.

## Abbreviations

MRF	magnetorheological figuring
SPDT	single-point diamond turning
CMP	chemical mechanical polishing
RMS	root mean square
Ra	surface roughness
PV	peak-to-valley
PSD	power spectral density
CCOS	computer-controlled optical surfacing
TIS	total integrated scattering
